# Assessment of Arterial Stiffness with Cardio-Ankle Vascular Index in Patients with Mitral Annular Calcification

**DOI:** 10.5152/eurasianjmed.2021.19235

**Published:** 2021-06

**Authors:** Faruk Boyaci, Murat Akcay, Engin Hatem, Ahmet Yanik, Tayyar Gokdeniz

**Affiliations:** 1 Clinic of Cardiology, Health Sciences University, Samsun Training and Research Hospital, Samsun, Turkey; 2 Department of Cardiology, Faculty of Medicine, Ondokuz Mayıs University, Samsun, Turkey; 3 Clinic of Cardiology, Mersin City Hospital, Mersin, Turkey; 4 Department of Cardiology, Medipol Mega Hospital, Medipol University, İstanbul, Turkey

**Keywords:** Vascular Stiffness, Mitral annular calcification, Atherosclerosis

## Abstract

**Objective:**

Arterial stiffness is related to arteriolosclerotic diseases and is a marker of adverse cardiovascular events. Mitral annular calcification (MAC) is progressive calcium deposition on the posterior and inferior mitral annulus and is associated with atherosclerotic cardiovascular diseases. Cardio-ankle vascular index (CAVI) is a measurement technique used to estimate the degree of arterial stiffness without effect from blood pressure. The aim of this study is to research arterial stiffness using CAVI in patients with MAC.

**Materials and Methods:**

The study was cross-sectional and observational and included 98 patients with MAC confirmed by echocardiography who referred to the cardiology clinics and met study inclusion criteria and 38 controls without MAC. CAVI measurements were obtained by using the Vascular Screening System VaSera VS-1000 (Fukuda Denshi, Tokyo, Japan) device.

**Results:**

The two groups were similar in terms of demographic characteristics, including age, sex, hypertension, coronary artery disease, body surface area, and smoking (*P* > .05). Left atrial volume index was significantly higher in patients with MAC compared with the control group (*P* < .001). Right arm CAVI, left arm CAVI, and mean CAVI were significantly higher in the MAC group than the control group (*P* = .037, *P* = .005, and *P* = .014, respectively) and increased with MAC severity. There was a significant positive correlation between mean CAVI and MAC grade (r = 0.278, *P* = .001). Also, when ankle-brachial index (ABI) was measured with CAVI, left and right extremity ABI values were significantly lower in patients with MAC (*P* = .017 and *P* = .005, respectively).

**Conclusion:**

CAVI increased in all patients with MAC and associated with increasing grade of calcification.

## Introduction

Mitral annular calcification (MAC) develops on progressive calcium deposition, usually on the entire mitral valve annulus, but particularly on the posterior and inferior regions. It is a chronic, non-inflammatory, and degenerative process. The frequency of MAC increases with age, and it tends to be more common and massive in women.^1^–^2^ Several studies demonstrated the association between MAC and different forms of atherosclerotic cardiovascular diseases, such as carotid stenosis, coronary artery disease, and aortic atheroma.^2^–^4^

Arterial stiffness is a marker of arteriosclerosis, develops as a result of thickening of the arterial wall and decreasing elasticity, and particularly involves the major arteries.^5^ Arterial stiffness is not only a marker of vascular aging but also a predictor of end-organ damage and a high frequency of cardiovascular events. It was previously demonstrated that arterial stiffness increases with aging and in the presence of cardiovascular or non-cardiovascular diseases, such as hypertension, atherosclerosis, diabetes, and rheumatoid arthritis.^5^ Cardio-ankle vascular index (CAVI) is a new measurement technique used to estimate the degree of arterial stiffness. The most significant advantage of CAVI is that it is not affected by blood pressure compared with other methods of measurement of arterial stiffness.^6^ Previous studies indicated that CAVI can be used for analyses of vascular functions, and it is related to coronary artery calcifications and atherosclerotic risk factors.^6^

In this study, we aimed to research arterial stiffness by using CAVI in patients with MAC.

## Materials and Methods

### Study Population

This study is a cross-sectional and observational design. The study population was formed from patients who were admitted to the cardiology clinics with different complaints and evaluated by echocardiography. Eligible patients who were informed about the study and ensured written informed consent were included in the study. The study protocol was approved by Kartal Koşuyolu Post Graduate Training and Research Hospital ethics committee (Ethic no: 2012.1/9) and adhered to the Declaration of Helsinki.

Patients confirmed as echocardiographically MAC-positive and MAC-negative formed the patient and control groups, respectively. A total of 136 subjects, including a patient group of 98 subjects who met the eligibility criteria and a control group of 38 subjects, were included. All individuals’ age, sex, body weight, height, cardiovascular risk factors, current medications, and history of other systemic diseases were recorded. The controls were selected according to the demographic characteristics of the MAC-positive group.

Patients with a rhythm other than sinus rhythm, poor image quality in echocardiography, ejection fraction (EF) < 50%, cardiomyopathy, previous myocardial infarction, moderate or severe valve insufficiency and obstruction, a prosthetic heart valve, rheumatic heart valve disease, chronic kidney failure (estimate glomerular filtration rate (eGFR) < 60 ml/dk/1.73 m^2^), cerebrovascular disease, peripheral arterial disease, and metabolic disorders such as diabetes mellitus and dyslipidemia were excluded from the study.

### Transthoracic Echocardiography and MAC

All patients were evaluated in echocardiography laboratories by using a Vivid S5 (GE Medical System, Horten, Norway; 3.5-MHz phased array transducer) echocardiography device. Echocardiographic images were performed while the patients were in lateral decubitus status. Parasternal long- and short-axis and apical four- and two-chamber views were obtained. M-mode, two dimensional (2-D), Doppler, and colored Doppler images and measurements were obtained and evaluated according to American Society of Echocardiography standards.

**Figure 1. a–c:**
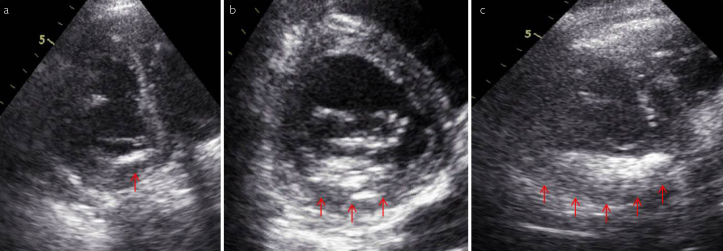
MAC positivity and echocardiographic grading. Grade 1 (mild, <3 mm thick, including less than one-third of the annulus), Grade 2 (moderate, 3–5 mm thick, less than two-thirds of the annulus), and Grade 3 (severe, >5 mm thick, intensively calcified annulus). MAC, mitral annular calcification.

MAC positivity was defined by the presence of bright echo-dense bands, located posterior to the mitral valve and progressing parallel to the left ventricular posterior wall in M-mode images, and posterior or inferior to the mitral leaflets in parasternal long-axis and apical four-chambered views and within the atrioventricular canal in parasternal short-axis views in 2-D echocardiographic images. In the parasternal short-axis cross-section images obtained at the level of the mitral valve, the region parallel to the myocardium located posterior to the annulus and inferior to the posterior leaflet was separated into 3 regions. MAC was classified according to the density of calcification as being Grade 1 (mild, <3 mm thick, including less than one-third of the annulus), Grade 2 (moderate, 3–5 mm thick, less than two-thirds of the annulus), and Grade 3 (severe, >5 mm thick, intensively calcified annulus) (Figure 1).^7^,^8^

EF and left ventricle volume were calculated by modified Simpson method. Left atrium volume was calculated by planimetric drawing of the left atrium areas (A1, A2) in apical four-chambered and two-chambered views and using the axial length of the left atrium, where at least 2 measurements were obtained from the apical four-chambered and two-chambered views and their average was used in calculations. Left atrium volume indices (LAVIs) were calculated based on the proportion of left atrial volume to body surface area.

### Arterial Blood Pressure and CAVI Measurements

Arterial blood pressure was calculated in compliance with World Health Organization guidelines by using a mercury sphygmomanometer (ERKA, Berlin, Germany) with a cuff suitable to the arm circumference and after resting for 20 minutes.

CAVI measurements were obtained at room temperature in a quiet environment by using a Vascular Screening System VaSera VS-1000 (Fukuda Denshi, Tokyo, Japan) device. Patients were asked to avoid eating and smoking for at least 3 hours before the measurements. Before the measurements, patients rested for 10 minutes in supine position. Parameters, including the patients’ name, age, height, and weight, were entered into the device. Four oscillometric measurement cuffs were used in total, 2 placed on the ankles and 2 on the arms. Cardiac rhythm and sounds were monitored by placing electrocardiogram electrodes on the right and left arms and a phonocardiogram on the second costal space to the right of sternum. Measurements were obtained after patients were asked to avoid speaking and moving during the test. The pressures and waveforms of brachial and ankle arteries were calculated, and pulse wave velocity (PWV) followed by CAVI were measured automatically. CAVI was calculated by the following calculation^6^,^9^,^10^:


$$\text{CAVI}=\text{a}[(2\text{r}/\Delta \text{P})\times \text{ln}(\text{Ps}/\text{Pd}){\text{PWV}}^{2}]+\text{b},$$

where Ps and Pd are systolic and diastolic blood pressure, respectively; PWV is from the origin of the aorta to the joint of the tibial artery with the femoral artery; ΔP is Ps − Pd, r is blood intensity, and a and b are constants. The equation was derived from Bramwell-Hill’s equation and the stiffness parameter β, and CAVI was regulated for blood pressure based on the stiffness parameter β. For this reason, CAVI projects the stiffness of the aorta, femoral artery, and tibial artery as a whole; classically, it is not influenced by blood pressure.^6^,^9^,^10^ After automatic calculations, data gathered were analyzed using VSS-10 software (Fukuda Denshi), and the values of right, left, and average of CAVIs were calculated.

Ankle-brachial index (ABI) was measured based on the systolic blood pressure (SBP) for both upper and lower extremities and then calculated by dividing the ankle SBP by the brachial SBP. Data collected were automatically analyzed using the VeSera data management software program (version: V10-01, Fukuda Denshi).^11^

### Statistical Analysis

Data obtained from the subjects were entered into SPSS v. 17.0 software for Windows (SPSS Inc.; Chicago, IL, USA). Continuous variables were reported as mean ± standard deviation, and categorical variables were reported as frequency and percentages. After the data were tested for normal distribution, Student’s *t* test was used to compare the mean values of continuous variables among 2 independent groups. Mean CAVI (M-CAVI) values were compared between the patient and control groups based on the grades of MAC by using one-way analysis of variance (ANOVA), and post-hoc analysis methods were applied to identify the source of difference. Pearson correlation coefficients were calculated to test the correlations between M-CAVI and all groups. The level of statistical significance was determined as *P* < .05.

## Results

A total of 136 subjects, with a mean age of 69.9 ± 7.9 years, were included in the study. The majority of the study population was women (n = 93, 68.4%). Comparison of the demographical, clinical, and echocardiographic data between patient and control groups did not indicate any significant difference in terms of age, gender, hypertension, coronary artery disease, body mass index, or smoking status (*P* > .05) (Table 1). Among the echocardiographic parameters, mitral inflow spectral Doppler E and A waves were significantly higher in the MAC-positive group (*P* < .05), and among the tissue Doppler parameters, mean mitral annulus myocardial e wave (E’m) was also significantly lower in the MAC-positive group (*P* < .05). LAVI was significantly higher in the MAC-positive group than the control group (26.43 ± 4.97 vs. 22.17 ± 5.66; *P* < .001) (Table 1). When ABI was measured with CAVI, left ABI (L-ABI) and right ABI (R-ABI) were significantly lower in MAC-positive patients than the control group (*P* = .017 and *P* = .005, respectively) (Table 1).

**Figure 2:**
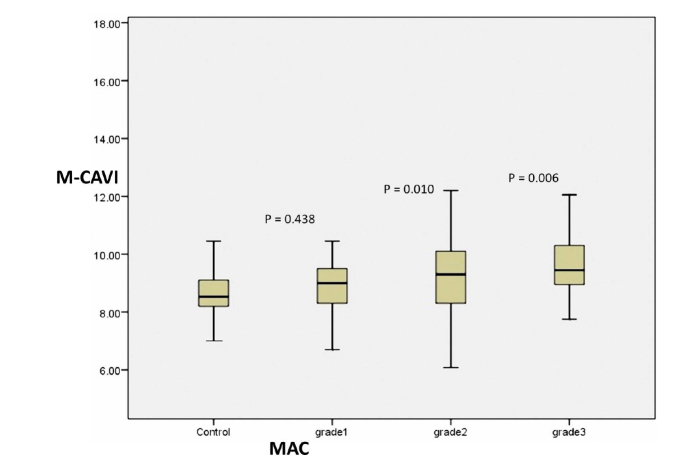
Box plot showing M-CAVI values of the control group and grade of MAC-positive groups. M-CAVI, mean cardio-ankle vascular index; MAC, mitral annular calcification.

Patients with MAC were divided into 3 groups as having Grade-1, Grade-2, and Grade-3 MAC based on the extent of calcification, and mean values for left CAVI (L-CAVI), right CAVI (R-CAVI), and M-CAVI were compared between these three groups and the controls. M-CAVI values of the control and MAC groups are shown in Figure 2. Table 2 shows the data obtained by ANOVA comparing the mean values between multiple independent groups. Table 2 shows that mean R-CAVI was significantly different between all MAC-positive groups and the control group (*P* = .037). Mean L-CAVI was also significantly different between all MAC-positive groups and the control group (*P* = .005). Similarly, M-CAVI was significantly different between all MAC-positive groups and the control group (*P* = .014).

**Figure 3:**
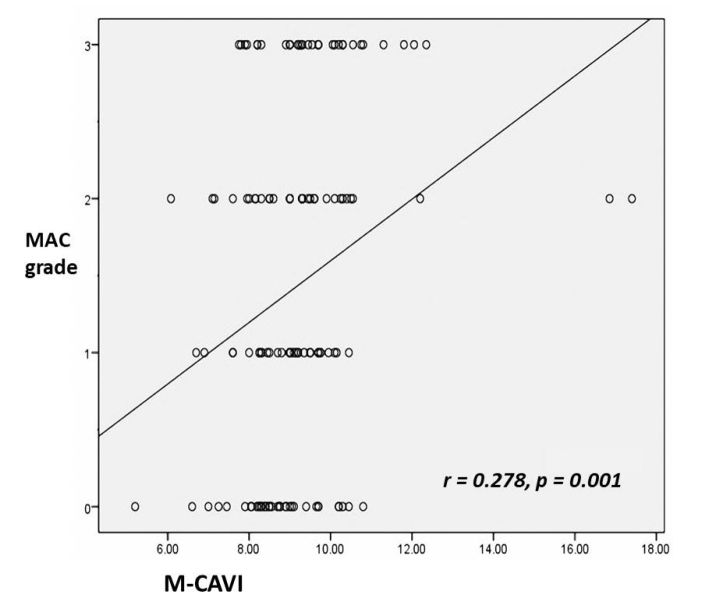
Graph of correlation analysis for MAC grades in all patients with M-CAVI. M-CAVI, mean cardio-ankle vascular index; MAC, mitral annular calcification.

Correlation analysis showed that there was a significant positive correlation between M-CAVI and MAC grades in the entire patient population (r = 0.278, *P* = .001) (Figure 3).

Post-hoc analyses were performed to identify which MAC grade groups were significantly different compared with the control group. Although L-CAVI was not found to be significantly different between the control and Grade-1 MAC groups (*P* = .293), L-CAVI in Grade-2 and Grade-3 MAC groups was significantly different compared with the control group (*P* = .006 and *P* = .002, respectively). Similarly, R-CAVI was not significantly different between the control and Grade-1 MAC groups (*P* = .625), but it was significantly different between Grade-2 and Grade-3 MAC groups and the control group (*P* = .016 and *P* = .031, respectively). M-CAVI in the control group was not significantly different compared with the Grade-1 MAC group (*P* = .438) but was significantly different from the Grade-2 (*P* = .010) and Grade-3 (*P* = .006) MAC groups (Table 3).

## Discussion

In this study, we found that arterial stiffness is increased in MAC-positive patients independent of the effect of blood pressure, although it was shown with indirect methods in previous studies. The results also show a novelty by establishing a positive association between MAC severity and arterial stiffness.

Cardiovascular calcification reflects pathological calcium phosphate deposition in blood vessels, the myocardium, and heart valves. Calcification of the heart valves was shown to be histopathologically similar to atherosclerotic plaques, supporting the opinion that calcification can be considered as a sign of atherosclerosis.^12^ MAC results from progressive calcium deposition on the entire mitral valve annulus, particularly on the posterior and inferior regions, and its prevalence was estimated as 14% in a subgroup analysis of the Framingham Heart Study including 1197 patients, whereas another study reported an incidence of 13%.^13^,^14^ When the mitral annulus, especially the posterior mitral leaflet, is exposed to high left ventricular systolic pressure, it leads to an increase in MAC frequency^7^. A previous study demonstrated that cardiovascular mortality increases by 10% with each 1-mm increase in calcification.^7^ The frequency of MAC reported by echocardiographic studies varies from 2.8% to 6.3% (7,15). MAC is more frequent in the elderly, women, and especially patients with renal failure.^7^,^15^,^16^ In our study, 72% of the MAC-positive group was female. The frequency of MAC was found to be 36% in a prospective study including patients with chronic renal failure.^16^

Previous studies demonstrated the relation between MAC and mitral stenosis, aortic annulus and leaflet calcification, and calcific aortic stenosis.^7^,^14^–^17^ Diastolic dysfunction is also more frequent in patients with MAC.^18^ Patients with MAC more frequently present with atrioventricular block, bundle block, and intraventricular conduction delay, and the relation between MAC and atrial fibrillation was demonstrated in a subgroup analysis of the Framingham Heart Study, which followed 1126 patients for 16 years (hazard ratio (HR), 1.6; 95% confidence interval (CI), 1.1–2.2).^14^,^15^,^19^ In our study, LAVI, a predictor of atrial fibrillation, was significantly elevated in patients with MAC. In the Strong Heart Study^20^, MAC was found to be an independent predictor of ischemic stroke, and it was significantly associated with stroke and carotid artery stenosis.^21^,^22^ MAC was associated with myocardial infarction, heart failure, and untoward cardiovascular events, including death.^22^

Increased arterial stiffness is a marker of endothelial dysfunction and a significant predictor of the progression of atherosclerosis, as well as adverse cardiovascular events, morbidity, and mortality.^23^ Arterial stiffness is effected by cardiovascular diseases and several risk factors, including age, smoking and long-term caffeine intake, obesity and dietary salt intake, a sedentary lifestyle, arterial hypertension, diabetes mellitus, hyperlipidemia, and metabolic syndrome.^23^,^24^ The relation of increased arterial stiffness with end-organ damage in vascular diseases, such as renal disease, stroke, myocardial infarction, and heart failure, and the clinical consequences of this relation are well-known. Assessment of arterial stiffness in routine clinical practice is significant to determine the progression of arteriosclerosis.^5^,^6^,^24^

CAVI was advanced to obtain measurements of arterial stiffness without being affected by blood pressure, and it reflects the stiffness of an artery over its entire length. Indeed, CAVI reflects the stiffness of the complete arterial system, from the tibial artery up to the femoral artery and the aorta.^6^ CAVI can be calculated based on the systolic and diastolic blood pressures measured above the level of the brachial artery and PWV measured from the tibial artery at ankle level to the aorta. This index is calculated by the use of the Bramwell-Hill equation and stiffness parameter β, suggested by Hayashi et al.^25^ and Kawasaki et al.^26^.

This was a unique study demonstrating the relation between MAC and arterial stiffness by using the CAVI method. Arterial stiffness was measured by CAVI, and M-CAVI was found to be significantly elevated in patients with MAC. R-CAVI, L-CAVI, and M-CAVI were significantly higher in the MAC group than the control group (*P* = .037, *P* = .005, and *P* = .014, respectively) and increased with MAC severity. Correlation analyses also showed that there was a significant, positive correlation between M-CAVI and MAC. Minoka et al.^27^ previously reported a significant relation between coronary artery calcification and CAVI in patients with diabetes. Korkmaz et al.^28^ found that arterial stiffness was significantly elevated in patients with asymptomatic aortic valve sclerosis by using CAVI. In another study performed by Durmus et al.^29^, PWV and arterial stiffness were found to be elevated in patients with MAC. In that study, PWV was measured by a sphygmocor device, which essentially depends on blood pressure, and augmentation index was not found to be significantly different. Although those results are in line with our findings, this study was carried out in a larger patient population and the increase in arterial stiffness was demonstrated by CAVI, which is a more objective method of measurement. In addition, CAVI is not a local stiffness parameter, but instead it projects the stiffness of all vessels.^9^ Recent studies have demonstrated that CAVI is correlated with subclinical parameters of atherosclerosis covering carotid intima media thickness and epicardial fat thickness.^30^,^31^

We also measured ABI with CAVI. ABI is a quick, non-invasive way to evaluate for peripheral artery disease. We found that ABI was lower in MAC-positive patients. In addition to evaluating arterial stiffness with CAVI, ABI is also evaluated in asymptomatic patients and information can be provided for pulmonary arterial hypertension. Our study is also important for demonstrating the reduction of ABI in asymptomatic MAC-positive patients.

Results of this study confirmed that arterial stiffness is increased in MAC-positive patients. The increase in arterial stiffness in patients with MAC was demonstrated to be independent of the effect of blood pressure in our study, although it was shown with indirect methods in previous studies. The results introduce a novelty by establishing an association between MAC severity and arterial stiffness.

### Study Limitations

This study included a small patient population, and the number of patients in the groups formed based on MAC grades was limited. Moreover, the design of this study focused on isolated MAC. Investigation of the relation between arterial stiffness and MAC, particularly in specific subgroups, will ensure a more detailed understanding on this subject.

In conclusion, we evaluated arterial stiffness with the CAVI method, which is an objective indicator of arterial stiffness in the complete arterial system. Arterial stiffness distinctly increased in MAC-positive patients and also correlated with grade of calcification.

**Main Points**

- Arterial stiffness is related to arteriolosclerotic diseases and is a marker of adverse cardiovascular events.
- Mitral annular calcification (MAC) is progressive calcium deposition on the posterior and inferior mitral annulus.
- Cardio-ankle vascular index (CAVI) is an objective measurement technique of arterial stiffness in the complete arterial system without effect from blood pressure.
- CAVI increased in all MAC (+) patients compared with control group and associated with increasing grade of calcification.

## Figures and Tables

**Table 1:** Basic Demographic Characteristics, Echocardiographic, and ABI Parameters in MAC-Positive and Control Group Patients

		MAC-negative control group (n = 38) (mean ± SD)	MAC-positive group (n = 98) (mean ± SD)	*P*-value
Age, years		68.87 ± 7.19	70.34 ± 8.17	.333
Gender, n (%)	Men	16 (42%)	27 (28%)	.103
	Women	22 (58%)	71 (72%)	
BSA, kg/m^2^		1.82 ± 0.15	1.76 ± 0.15	.071
Systolic BP, mmHg		135.5 ± 12.41	138.5 ± 15.41	.052
Diastolic BP, mmHg		84.7	86.07	.07
Cigarette, n (%)		2 (5%)	7 (7%)	.693
Hypertension, n (%)		28 (74%)	75 (76%)	.729
CAD, n (%)		2 (5%)	13 (13%)	.183
EF, %		60.84 ± 3.97	60.27 ± 4.96	.527
E wave velocity, m/s		0.63 ± 0.14	0.72 ± 0.23	.006
A wave velocity, m/s		0.74 ± 0.22	0.98 ± 0.22	<.001
S’m wave velocity, cm/s		8.07 ± 2.16	7.81 ± 1.80	.473
E’m wave velocity, cm/s		8.10 ± 2.84	7.01 ± 1.79	.032
A’m wave velocity, cm/s		10.26 ± 2.61	10.93 ± 2.72	.192
LAVI, mL/m^2^		22.17 ± 5.66	26.43 ± 4.97	<.001
R-ABI		1.12 ± 0.09	1.07 ± 0.10	.005
L-ABI		1.11 ± 0.09	1.05 ± 0.14	.017

A wave velocity, mean peak late filling wave velocity due to atrial contraction; A’m wave velocity, mean peak late diastolic annulus velocity; ABI, ankle-brachial index; BP, blood pressure; BSA, body surface area; CAD, coronary artery disease; E wave velocity, mean peak early filling wave velocity; E’m wave velocity, mean peak early diastolic annulus velocity; EF, ejection fraction; L-ABI, left ankle-brachial index; LAVI, left atrial volume index; MAC, mitral annular calcification; S’m wave velocity, mean peak systolic annulus velocity; R-ABI, right ankle-brachial index. (Important P-values are in boldface).

**Table 2:** Right, Left, and Mean CAVI According to MAC-Positive Grades and Control Group

	Control (n = 38)	Grade-1 (n = 33)	Grade-2 (n = 34)	Grade-3 (n = 31)	*P*-value
R-CAVI	8.74 ± 1.07	8.91 ± 0.94	9.55 ± 2.16	9.49 ± 1.11	.037
L-CAVI	8.52 ± 1.24	8.92 ± 0.89	9.55 ± 2.30	9.74 ± 1.45	.005
M-CAVI	8.63 ± 1.09	8.90 ± 0.89	9.53 ± 2.25	9.61 ± 1.24	.014

CAVI, cardio-ankle vascular index; L-CAVI, left cardio-ankle vascular index; M-CAVI, mean cardio-ankle vascular index; R-CAVI, right cardio-ankle vascular index.

**Table 3:** Results of Post-Hoc Analysis to Determine the Differences Between Control and MAC-Positive Groups Originated From MAC Grades

	P-Value		

	L-CAVI control group	R-CAVI control group	M-CAVI control group
MAC-positive, Grade 1	.293	.625	.438
MAC-positive, Grade 2	.006	.016	.010
MAC-positive, Grade 3	.002	.031	.006

L-CAVI, left cardio-ankle vascular index; M-CAVI, mean cardio-ankle vascular index; MAC, mitral annular calcification; R-CAVI, right cardio-ankle vascular index.
